# IL-23R is Epigenetically Regulated and Modulated by Chemotherapy in Non-Small Cell Lung Cancer

**DOI:** 10.3389/fonc.2013.00162

**Published:** 2013-06-19

**Authors:** Anne-Marie Baird, Éilis Dockry, Anne Daly, Emma Stack, Derek G. Doherty, Kenneth J. O’Byrne, Steven G. Gray

**Affiliations:** ^1^Department of Clinical Medicine, Trinity College Dublin, Dublin, Ireland; ^2^Thoracic Oncology Research Group, Institute of Molecular Medicine, St. James’s Hospital, Dublin, Ireland; ^3^Department of Immunology, Trinity College Dublin, Dublin, Ireland; ^4^HOPE Directorate, St. James’s Hospital, Dublin, Ireland; ^5^Cancer Services, Princess Alexandra Hospital, Cancer and Ageing Research Program, Queensland University of Technology, Brisbane, Australia

**Keywords:** IL-23R, non-small cell lung cancer, epigenetics, acetylation, methylation, apilimod

## Abstract

The Interleukin-23 (IL-23)/IL-23R signaling axis is an important inflammatory pathway, involved in the stimulation and regulation of the T helper (Th) 17 lymphocytes, resulting in the production of IL-17. Aside from auto-immunity, this cytokine has also been linked to carcinogenesis and polymorphisms in the IL-23R gene are associated with an increased risk for the development of a number of different cancers. Activation of the IL-23 pathway results in the up-regulation of STAT3 and it is thought that the pathological consequences associated with this are in part due to the production of IL-17. We have previously identified IL-23A as pro-proliferative and epigenetically regulated in non-small cell lung cancer (NSCLC). The current study aims to evaluate IL-23R in greater detail in NSCLC. We demonstrate that IL-23R is expressed and epigenetically regulated in NSCLC through histone post-translation modifications and CpG island methylation. In addition, Gemcitabine treatment, a chemotherapy drug used in the treatment of NSCLC, resulted in the up-regulation of the IL-23R. Furthermore, Apilimod (STA 5326), a small molecule which blocks the expression of IL-23 and IL-12, reduced the proliferative capacity of NSCLC cells, particularly in the adenocarcinoma (A549) sub-type. Apilimod is currently undergoing investigation in a number of clinical trials for the treatment of auto-immune conditions such as Crohn’s disease and Rheumatoid Arthritis. Our results may have implications for treating NSCLC patients with Gemcitabine or epigenetic targeted therapies. However, Apilimod may possibly provide a new treatment avenue for NSCLC patients. Work is currently ongoing to further delineate the IL-23/IL-23R axis in this disease.

## Introduction

Lung cancer is a worldwide health burden, with numbers increasing both in terms of incidence and mortality. In 2008, there was an estimated 1.61 million cases of lung cancer, which accounted for 12.7% of total cancer incidence, while in Europe an estimated 410,000 cases of lung cancer occurred in 2012 (Ferlay et al., 2013). In terms of mortality, lung cancer accounted for 18.2% of all cancer related deaths, which is equivalent to 1.38 million people (Ferlay et al., 2010). In Europe an estimated 353,000 deaths occurred in 2012 for lung cancer (Ferlay et al., 2013). Due to the difficulties in early detection, combined with fact that the majority of patients present at an advanced stage, lung cancer has a high morbidity, 5-year survival rates continue to remain poor, at approximately 6–14% in males and 7–18% in females (Youlden et al., 2008). Recently lung cancer has overtaken breast and prostate as the biggest cancer killer in females and males respectively in a number of countries.

Inflammation plays an important role in lung carcinogenesis (O’Callaghan et al., 2010), given that individuals with inflammatory lung conditions have an increased risk of lung cancer development. This risk can persist even in the absence of tobacco use, further underscoring the role of chronic inflammation in lung carcinogenesis (Demaria et al., 2010; Mortimer et al., 2012). Due to the massive global burden of lung cancer in both smoking and non-smoking individuals and in particular the dramatic rise in the disease in never smokers, there is an urgent need to identify both new biomarkers in this disease and novel druggable targets to improve treatment options and increase survival rates. To this end inflammatory mediators may provide a significant innovative therapeutic avenue in this disease (Demaria et al., 2010).

We have previously discussed the potential role of epigenetics in the setting of lung disease (Lawless et al., 2009, 2010). Indeed aberrant epigenetic events are frequent in cancer (You and Jones, 2012), including lung cancer (Balgkouranidou et al., 2013). Epigenetic targeting of lung cancer continues to be actively researched (Juergens et al., 2011; Tsai et al., 2012). Within this context we have shown that many pro-inflammatory cytokines and genes associated with pro-inflammatory cues are epigenetically regulated and can be targeted in lung cancer using epigenetic targeting agents (Baird et al., 2011a,b; Cathcart et al., 2011). Most recently we have shown that Interleukin-23 (IL-23) as being pro-proliferative and epigenetically regulated in non-small cell lung cancer (NSCLC) (Baird et al., 2013).

Interleukin-23 and IL-12 are members of a small family of pro-inflammatory hetero-dimeric cytokines and are part of the wider IL-6 superfamily (Croxford et al., 2012; Vignali and Kuchroo, 2012). Both cytokines share a common p40 sub unit that is covalently linked either to a p35 subunit to form IL-12 or to a p19 sub unit to form IL-23 (Oppmann et al., 2000). IL-12 was considered as the cytokine responsible for a number of auto-immune diseases such as experimental auto-immune encephalomyelitis (EAE), an animal model of Multiple Sclerosis. However, knockout murine studies demonstrated that this was not the case, as p35^−*/*−^ mice were susceptible to EAE but p40^−*/*−^ mice were resistant (Becher et al., 2002). This was confirmed in p19^−*/*−^ mice, where it was shown that p19 was essential for the development of EAE (Cua et al., 2003).

Activated Dendritic Cells (DC) and macrophages are both sources of IL-23 (Croxford et al., 2012). When stimulated by IL-23, macrophages produce TNF-α, IL-1, and IL-23 itself (Duvallet et al., 2011). IL-23 promotes inflammatory responses such as in the defense against bacterial infection, up-regulation of angiogenic factors and MMPs (Langowski et al., 2006) and drives an IL-17 producing T cell population (Th 17) in part through the activation of the STAT3 pathway (Langowski et al., 2006; Harris et al., 2007). It has been shown that IL-23 provides a connection between the pro-inflammatory processes which promote tumor growth and the failure of adaptive immune mediators to penetrate tumors (Langowski et al., 2006; Langowski et al., 2007). As such targeting IL-23 mediated Th17 responses may have important implications in the treatment of pro-inflammatory conditions including cancer (Miossec and Kolls, 2012).

The receptor, through which IL-23 elicits its effects, IL-23R, is composed of an IL-12Rβ1, a subunit common to the IL-12 receptor, and an IL-23R chain (Duvallet et al., 2011). Monocytes, DC, natural killer cells, natural killer T cells, and myeloid cells, amongst others, express IL-23R (Parham et al., 2002; Duvallet et al., 2011). Furthermore, polymorphisms in the IL-23R have been linked to a number of cancers, including gastric (Chen et al., 2010), colorectal (Poole et al., 2012), oral (Chien et al., 2012), esophageal (Chu et al., 2012), leukemia (Qian et al., 2013a), hepatitis B virus (HBV)-related hepatocellular carcinoma (Xu et al., 2013), breast, lung, and nasopharyngeal cancer (Zheng et al., 2012). In IL-23R knockout mice, the tumor growth of both B16F10 melanoma and LL2 lung carcinoma were inhibited in the IL-23R^−*/*−^ mice (Langowski et al., 2006). Expression of the IL-23R is associated with pro-inflammatory cues as it can be regulated by various cytokines including TNF-α (Charles et al., 2009), IL-6 (Zhou et al., 2007), IL-21, and IL-23 itself (Ivanov et al., 2007). Elevated levels of IL-23R receptor expression have also been demonstrated in ovarian cancer (Charles et al., 2009), NSCLC (Li et al., 2013) and in colorectal cancer (Lan et al., 2011; Suzuki et al., 2012).

As such, the IL-23/IL-23R axis is emerging as an important potential therapeutic pathway in both auto-immune conditions and the carcinogenic process. Following from this, a small molecule inhibitor has been developed called Apilimod mesylate (STA 5326) that selectively inhibits the production of the cytokines, IL-12 and IL-23 (Wada et al., 2007), and is currently undergoing evaluation in a number of clinical trials in auto-immune conditions (Billich, 2007; Wada et al., 2012).

We and others have previously identified IL-23 as pro-proliferative (Baird et al., 2013; Li et al., 2013), and epigenetically regulated in NSCLC (Baird et al., 2013). In this study we therefore sought to examine the role of IL-23R in NSCLC in greater detail, and to determine the effects of Apilimod on NSCLC cells. We show that similar to what we have previously observed for IL-23A, expression of IL-23R is also epigenetically regulated, and affected by chemotherapeutic agents. We also show that targeting IL-12/IL-23 production using Apilimod results in significant anti-proliferative responses in NSCLC cell lines, further underlining the therapeutic potential of targeting this pathway in cancer.

## Materials and Methods

### Cell lines

The A549 (adenocarcinoma) and SK-MES-1 (squamous cell carcinoma) were purchased from the ATCC (LGC Promochem, Teddington, UK). All cell culture reagents were purchased from Lonza (Walkersville, MD, USA) unless stated otherwise. Cells were maintained at 37°C in a humidified atmosphere containing 5% CO_2_ in the following media; A549 – F-12 (Ham) medium supplemented with 10% (v/v) FBS, penicillin streptomycin (500 U/mL), and 2 mM l-glutamine. SK-MES-1 – EMEM with the addition of 10% (v/v) FBS, penicillin streptomycin (500 U/mL), 2 mM l-glutamine, and 0.1 M non-essential amino acids.

### Primary tumor samples

A series of 37 tumor specimens (21 adenocarcinoma, 16 squamous cell carcinoma) were taken from patients presenting with early stage NSCLC at either St. James’s Hospital, Dublin, Ireland or were obtained from the Leicester Royal Infirmary lung cancer biobank, Leicester, UK. Matched normal tissue was taken in parallel for each patient and samples were evaluated by a pathologist immediately following dissection. Informed consent was obtained from each patient, and the study was conducted after formal approval from local Hospital Ethics Committees. We did not generate any primary tumor derived cell lines from the resected material.

### Reagents

TrichostatinA (TSA) was purchased from Calbiochem (San Diego, CA, USA) and dissolved in DMSO. Cell cultures were treated for a period of 24 h, at a final concentration of 250 ng/mL.

Suberoylanilide Hydroxamic Acid (SAHA) was obtained from Cayman Chemical (Ann Arbor, MI, USA) and dissolved in DMSO. Cell cultures were treated for 24 h, at a final concentration of 5 μM).

5-Aza-2′-Deoxycytidine (DAC) was purchased from Merck (Darmstadt, Germany) and dissolved in methanol. Cell cultures were treated with DAC (final concentration – 0.2 or 1 μM) for 48 h with DAC and media replaced every 24 h.

2′-Deoxy-2′,2′-difluorocytidine hydrochloride (Gemcitabine – GEM) was supplied by Eli Lilly (Indianapolis, IN, USA) and dissolved in PBS at a final concentration of 38 mg/mL (120.14 mM). Cell cultures were treated with GEM (final concentration – 0.2 or 1 μM) for 48 h with GEM with media and drug replaced every 24 h.

Apilimod (STA 5326) was purchased from Axon Medichem BV (Groningen, Netherlands). Cells were treated at various concentrations ranging from 0.1 μM to 1 mM for 72 h.

### RNA isolation and RT-PCR amplification

Total RNA was extracted using TRI reagent ®(MRC, Cincinnati, OH, USA) according to the manufacturer’s instructions. Prior to first strand cDNA synthesis, 1 μg of total RNA was pre-treated by digestion with RQ1 DNase (Promega, Madison, WI, USA) according to the manufacturer’s instructions. cDNA was generated using RevertAid (Fermentas, St. Leon-Rot, Germany) and OligodT(20) primers according to the manufacturer’s instructions.

Cell lines were examined for the expression of *IL-23R* (171 bp) (Forward 5′-CAGGTCACTATTCAATGGGATGC-3′, Reverse 5′-GCAGTTCTTAATTGCTGCTTGG-3′) and *Beta-actin* (510 bp) (Forward 5′-AGCACTGTGTTGGCGTACAG-3′, Reverse 5′-TGTTTGAGACCTTCAACACCC-3′) by RT-PCR. Cycling conditions consisted of: 95°C for 5 min followed by 35 cycles of 1 min at 94°C, 1 min at the target gene annealing temperature (IL-23R – 58°C, Beta-actin – 55°C) and 1 min at 72°C with a final extension at 72°C for 10 min.

To examine IL-23R expression in primary patient material, we compared *IL-23R* mRNA levels in the tumor tissue to matched normal tissue from the same individual as follows. The samples were subjected to RT-PCR for *IL-23R* and *Beta-Actin*, and electrophoresed on 2% agarose gels. If there was a distinct (visible) change in expression samples were classified as either up/down/unchanged.

Subsequently we conducted “semi-quantitative” densitometric analysis on the gene expression. *IL-23R* expression was normalized to the *Beta-Actin* loading control which resulted in a discrete ratio value. The values for all samples were averaged and a Student’s *t-*test was performed to compare Normal versus Tumor *IL-23R* expression. No cut off points were used and all data is based on the average values obtained.

Experiments conducted on cell lines were carried out in triplicate and PCR products electrophoresed on a 2% agarose gel. Product quantification was performed using TINA 2.09c (Raytest, Isotopenmeßgeräte GmbH, Straubenhardt, Germany) densitometry software. The target mRNA expression was normalized to Beta-actin controls, and was expressed as a ratio of target mRNA expression: *Beta-actin* expression.

### Chromatin immunoprecipitation

Chromatin immunoprecipitation (ChIP) was performed as follows: following treatments, cells were fixed with formaldehyde (final concentration 1%), suspended in SDS lysis buffer (Millipore, Billerica, MA, USA) and sonicated until DNA was fragmented into lengths of between 200 and 1000 bp. Aliquots of this sheared DNA were subsequently immunoprecipitated using the OneDayChIP Kit ™(Diagenode, Liege, Belgium) according to the manufacturer’s instructions.

The antibodies used for immunoprecipitation were as follows: pan acetyl-histone H3 (H3Ac) (Millipore, Cat#06-599), pan acetyl-histone H4 (H4Ac) (Millipore, Cat#06-598), acetyl-histone H3 Lys 9/14 (H3K9/14ac) (Diagenode, Cat#pAb-ACHBHS-044), acetyl-histone H3 Lys 9 (H3K9Ac) (Diagenode, Cat#pAb-ACHAHS-044), di methyl-histone H3 Lys 9 (H3K9Me2) (Sigma, Cat#D5567), di methyl-histone H3 Lys 4 (H3K4Me2) (Sigma, Cat#D5692), methyl-histone H3 Lys 4 (H3K4Me) (Sigma, Cat#M4819), and acetyl-histone H3 Lys 9 phosphoSer10 (H3K9S10) (Sigma, Cat#H0788). A no antibody control was included to test for non-specific binding.

Primers used to study the promoter region of *IL-23R* by ChIP (172 bp) were designed from the promoter sequence at the Transcriptional Regulatory Element Database (http://rulai.cshl.edu/TRED) (Jiang et al., 2007), (Forward 5′-TTCTGCCTCTTGGATGAGACC-3′, Reverse 5′-CAGAGCCCTGACCTACATTGC-3′). PCR cycling conditions consisted of: 95°C for 5 min followed by 35 cycles of 1 min at 94°C, 1 min at the 58°C, and 1 min at 72°C with a final extension at 72°C for 10 min.

### Proliferation assay

Cell proliferation was measured using a Cell Proliferation ELISA, BrdU (Roche Diagnostics Ltd., Sussex, UK). Briefly, cells were seeded at 5^3^ × 10^3^/well in a 96-well plate and adhered overnight. Subsequently the complete media was removed and the cells washed with 100 μL PBS. Serum depleted media (0.5% FBS) was added, as this mimics more closely physiological conditions. Inhibition studies were carried out by treating cells with various concentrations of Apilimod (0.1 μM–1 mM) for 72 h. Absorbance was measured on a plate reader at 450 nm with a reference wavelength set to 690 nm. Blank and untreated (UT) wells were used for normalization purposes. The UT cells were set as 100%, and the Apilimod treatments assessed relative to this.

### Statistical analysis

The data are expressed as mean ± SEM. Statistical analysis was performed with Graphpad Prism 5.01 (Graphpad Software, La Jolla, CA, USA) using either Student’s *t*-test or a one-way analysis of variance (ANOVA) where groups in the experiment were three or more. Following ANOVA a *post hoc* test was performed using Dunnett’s Multiple Comparison Test. Differences were considered significant when *p* < 0.05.

## Results

### *IL-23R* is expressed in primary NSCLC specimens

**Figure 1:**
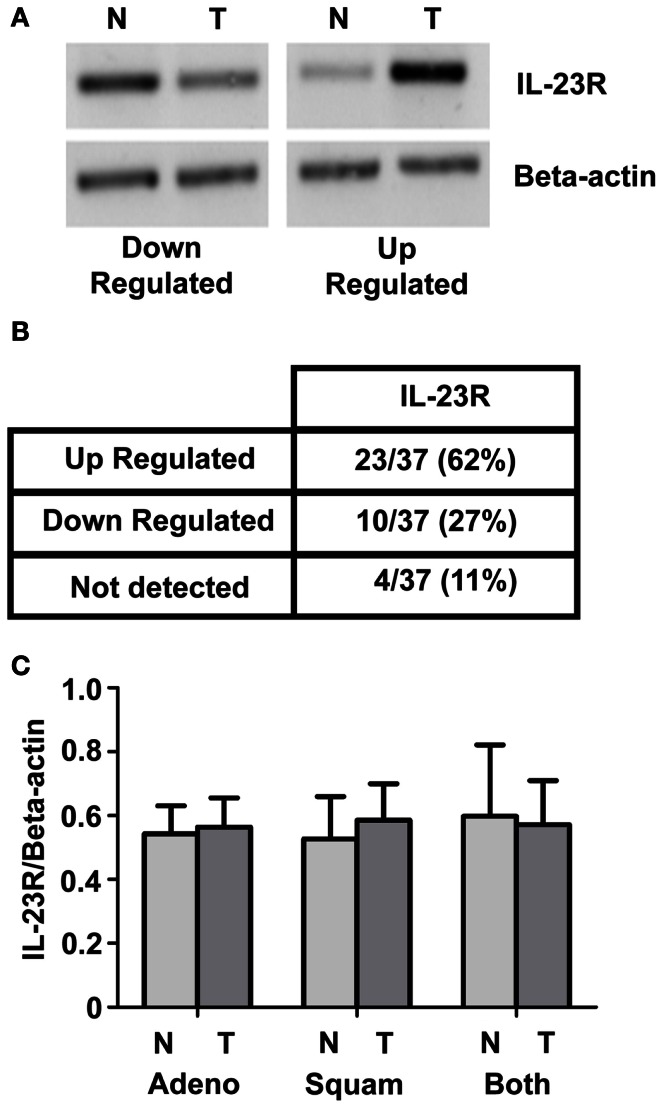
***IL-23R* mRNA expression in normal/tumor NSCLC matched pairs**. **(A)** Levels of *IL-23R* were examined by RT-PCR on a panel of 37 NSCLC [adenocarcinoma (*n* = 21) and squamous cell carcinoma (*n* = 16)] patient samples. Representative images of up and down regulated samples are shown. **(B)** A summary of the changes in expression of *IL-23R* in a panel of tumor/normal matched patient NSCLC samples. **(C)** Overall densitometry analyses of tumor/normal matched patient NSCLC samples. *Beta-actin* levels were used for normalization purposes. Data is expressed as mean ± SEM (*n* = 37). Statistical analysis was performed using a paired one-tailed Student’s *t*-test (N, normal; T, tumor; Adeno, adenocarcinoma; Squam, squamous cell carcinoma; Both – combined Adeno and Squam).

*IL-23R* expression was examined in a panel of normal/tumor matched NSCLC patient samples using RT-PCR Representative images are shown in Figure 1A. A summary of the results is shown in Figure 1B. Overall, there was an increase observed in *IL-23R* (23/37, 62%) in the tumor compared with normal. This did not reach significance for either the overall or separated cohorts of patient samples. Graphed densitometry analysis is shown in Figure 1C.

### *IL-*23R expression is epigenetically regulated through histone acetylation

**Figure 2:**
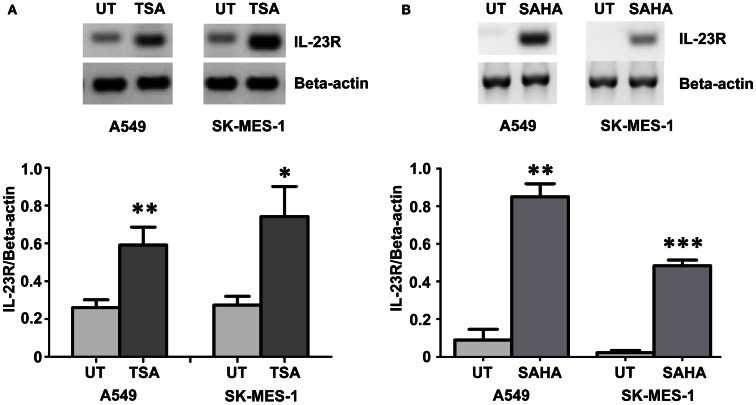
**Histone deacetylase inhibitors (HDi) induce *IL-23R* expression in NSCLC cell lines**. **(A)** Treatment with TSA (250 ng/mL) induced the expression of *IL-23R* in the A549 and SK-MES-1 cell lines. **(B)** Induction of *IL-23R* was also observed in both cell lines with SAHA treatment (5 μM). Densitometry analysis of expression in treated versus untreated samples when normalized to *Beta-actin*. Data is graphed as mean ± SEM (*n* = 3). Statistical analysis was performed using an unpaired Student’s *t*-test (*p < 0.05; **p < 0.01; ***p < 0.001) (UT, untreated; TSA, Trichostatin A; SAHA, Suberoylanilide Hydroxamic Acid).

Using the histone deacetylase inhibitor (HDi), Trichostatin A (TSA), significant induction of *IL-23R* was observed in both the A549 and SK-MES-1 cell lines (Figure 2A) (*p* < 0.05). This induction of *IL-23R* expression indicates that this gene is epigenetically regulated at the level of histone acetylation. Confirmation of this was obtained using treatment with the FDA approved HDi, SAHA (Vorinostat), which also resulted in increased *IL-23R* expression in both of the NSCLC cell lines examined (Figure 2B) (*p* < 0.05).

### Regulation of *IL-23R* occurs through direct chromatin remodeling

**Figure 3:**
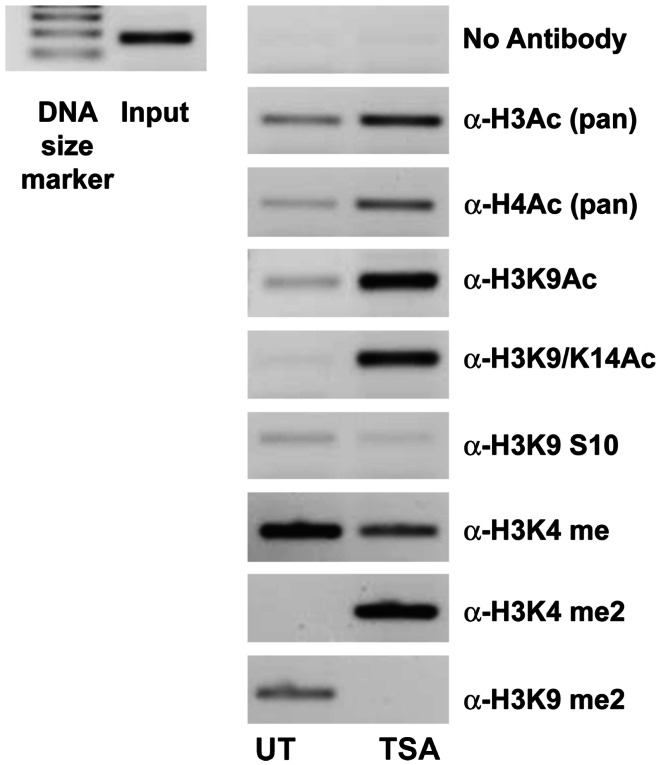
**Histone acetylation occurs directly at the promoter region of *IL-23R***. The ChIP assay demonstrates that TSA treatment results in an increase in the acetylation of histone H3 and H4. A549 cells were cultured in the presence or absence of TSA (250 ng/mL) for a period of 24 h. Subsequently, a ChIP assay was performed using the following antibodies; pan acetyl-histone H3 (H3Ac), pan acetyl-histone H4 (H4Ac), acetyl-histone H3 Lys 9 (H3K9Ac), acetyl-histone H3 Lys 9/14 (H3K9/14ac), acetyl-histone H3 Lys 9 phosphoSer10 (H3K9S10), methyl-histone H3 Lys 4 (H3K4Me), di methyl-histone H3 Lys 4 (H3K4Me2), and di methyl-histone H3 Lys 9 (H3K9Me2). Input DNA serves as a positive control as recommended by the manufacturer (Diagenode). A no antibody control was included to test for non-specific binding (UT, untreated; TSA, Trichostatin A).

A ChIP analysis of the IL-23R promoter from A549 cells pre-treated with TSA was used to verify that the observed effects for HDi were due to increased histone hyperacetylation. As shown in Figure 3, clear enrichment of histone acetylation was observed at the *IL-23R* promoter in response to TSA, indicated by an increase in the amount of PCR product for acetylated Histone H3 and H4. We show that lysine 9 and lysine 14 are also hyperacetylated in the *IL-23R* promoter region. Additional evidence that *IL-23R* is dynamically regulated by histone post-translational modifications is observed by an increase in histone H3 lysine 4 di methylation (H3K4me2) marker. The ChIP analysis confirms that chromatin remodeling is directly involved with the induction of *IL-23R* gene expression.

### *IL-23R* is also regulated through DNA CpG methylation

**Figure 4:**
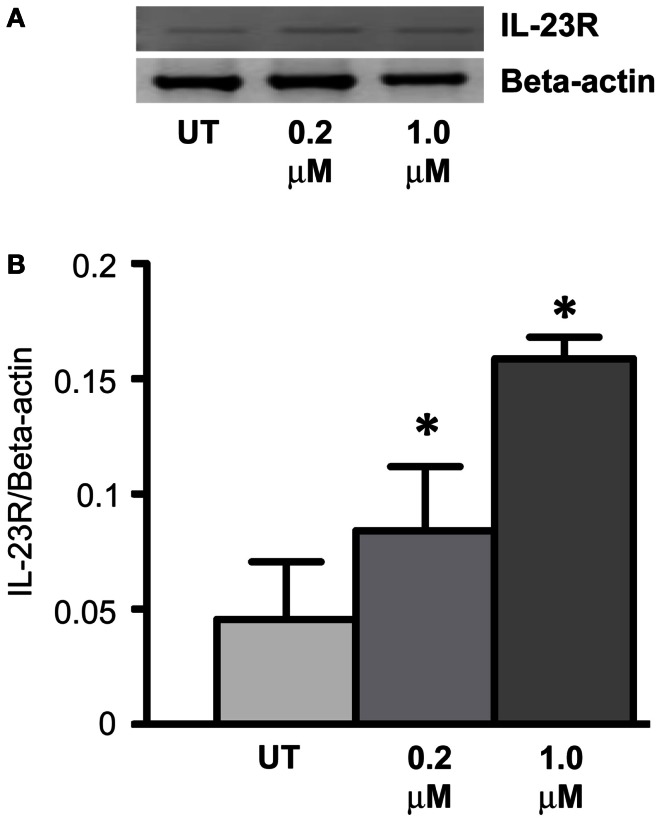
**Cell line response to a DNA methyltransferase inhibitor (DNMTi)**. **(A)** The effect of 5-aza-2′deoxycytidine (DAC) treatment on the expression of *IL-23R*. A549 cells were cultured in either 0.2 or 1.0 μM DAC for 48 h with media and drug replaced every 24 h. A PCR was also carried out for *Beta-actin* to determine loading efficiency and for normalization purposes. **(B)** Densitometry analysis of expression in treated versus untreated samples when normalized to *Beta-actin*. Data is graphed as mean ± SEM (*n* = 3). Statistical analysis was performed using an unpaired Student’s *t*-test (*p < 0.05) (UT, untreated; DAC, 5-aza-2′deoxycytidine).

Expression of *IL-23R* was examined in the A549 cells post-treatment with a DNA methyltransferase inhibitor (5-Aza-2′-Deoxycytidine – DAC) for 48 h (Figure 4A). DAC treatment significantly increased the mRNA expression of *IL-23R* at both concentrations tested (0.2, 1 μM, *p* < 0.05) in the A549 cells (Figure 4B). These results would suggest that the partial methylation of CpG islands in the *IL-23R* promoter is important in the regulation of this gene.

### Gemcitabine treatment results in increased expression of *IL-23R*

**Figure 5:**
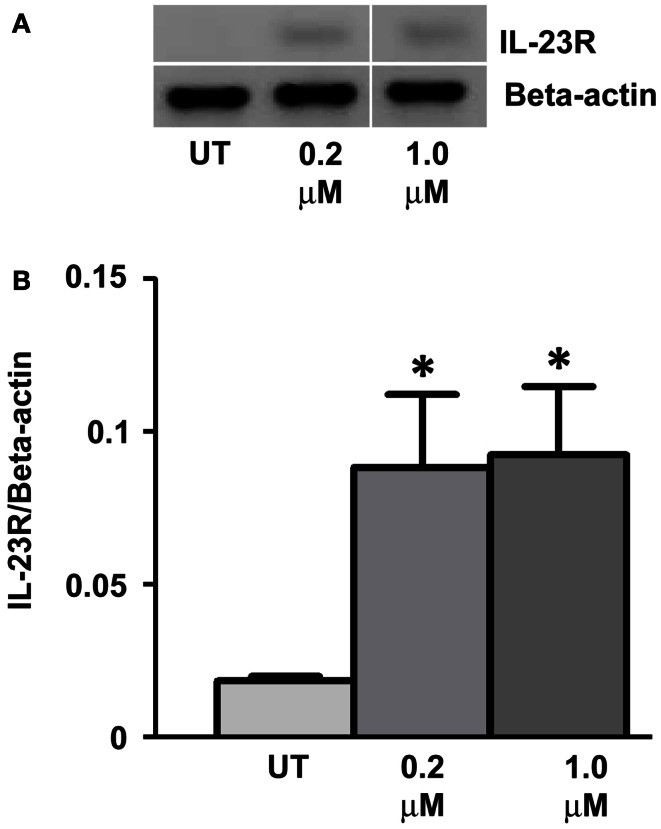
**Treatment with the chemotherapy drug, Gemcitabine, induces the expression of *IL-23R***. **(A)** The effect of Gemcitabine treatment on the expression of *IL-23R*. A549 cells were cultured in either 0.2 or 1.0 μM Gemcitabine for 48 h with media and drug replaced every 24 h. A PCR was also carried out for *Beta-actin* to determine loading efficiency and for normalization purposes. **(B)** Densitometry analysis of expression in treated versus untreated samples when normalized to *Beta-actin*. Data is graphed as mean ± SEM (*n* = 3). Statistical analysis was performed using an unpaired Student’s *t*-test (*p < 0.05) (UT, untreated; GEM, gemcitabine).

Gemcitabine (GEMZAR ® – Eli Lilly and Company) in combination with cisplatin is FDA approved for the first-line treatment of patients with NSCLC. A549 cells were treated with Gemcitabine (Gray et al., 2012) for a period of 48 h with drug and media replaced every 24 h. RT-PCR was utilized to determine changes in *IL-23R* expression (Figure 5A). Similar to the results observed with DAC, GEM treatment significantly increased the expression of *IL-23R* at concentrations of 0.2 and 1 μM (Figure 5B) (*p* < 0.05).

### Apilimod (STA 5326) decreases the proliferative capacity of NSCLC cells

**Figure 6:**
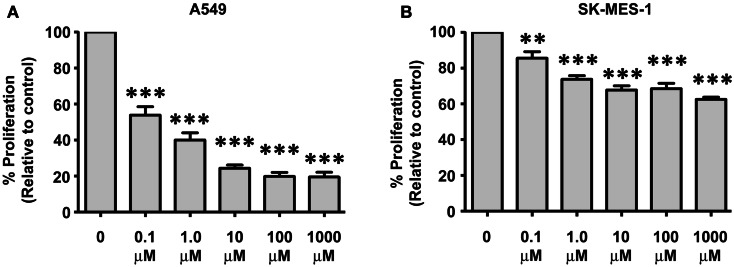
**Decreases in NSCLC cellular proliferation are observed post-Apilimod (STA 5326) treatment**. Cell proliferation was examined by a Cell Proliferation ELISA following 72 h of stimulation with Apilimod (0.1 μM–1 mM) in the **(A)** A549 and **(B)** SK-MES-1 cell lines. All treatment doses resulted in marked decreased proliferation in the A549 cells when compared with the SK-MES-1 cells. Data is represented as a percentage of the untreated control (UT), which was set to 100% and is expressed as mean ± SEM (*n* = 3). Statistical analysis was performed using a One-way ANOVA and a *post hoc* Dunnett’s Multiple Comparison Test (***p < 0.001; **p < 0.01).

We have previously shown that IL-23 is pro-proliferative in NSCLC (Baird et al., 2013). The A549 and SK-MES-1 cell lines were treated with various doses of Apilimod (0.1, 1, 10, 100 μM, and 1 mM) for 72 h and proliferation measured using a Cell Proliferation ELISA (Figure 6). All five doses of compound significantly reduced the proliferative capacity of both cell lines (Figure 6). However, the A549 cell lines demonstrated increased sensitivity to Apilimod (Figure 6A) compared with the SK-MES-1 cell line (Figure 6B). For instance even at the lowest concentration at 0.1 μM the proliferative rate in the A549 cell line (compared with UT) was reduced by almost 50% (53.94 ± 4.72%) in contrast to the SK-MES cell line (compared with UT) which was reduced by approximately 15% (85.6 ± 3.57%). At the highest concentration examined (1 mM), proliferation reduced by 40% in the squamous cell line (62.57 ± 1.25%), however in the adenocarcinoma cell line the rate decreased by 80% (19.56 ± 2.63%). These marked differences between the cell lines most likely reflect the contrast observed previously with recombinant IL-23 treatment (Baird et al., 2013), which was confirmed by Ping Lin and colleagues (Li et al., 2013). As such differential receptor expression may play a role in lung cancer cellular response to Apilimod.

## Discussion

This study builds on previous work undertaken in our laboratory, where we demonstrated increased expression of IL-23 in NSCLC and the pro-proliferative effect of recombinant IL-23 treatment (Baird et al., 2013). These results were independently confirmed in a separate study carried out by Li et al. (2013). We were the first to establish that IL-23 was subject to epigenetic regulation in NSCLC. We therefore wished to examine the IL-23/IL-23R axis in further detail in NSCLC given the involvement of this pathway in airway inflammation, and its potential therapeutic utility (Miossec and Kolls, 2012).

The expression of the IL-23R was determined in a panel of 37 NSCLC normal tumor matched patient tissues. Although an overall increase in expression was observed in tumor compared with normal (Figure 1B), this did not reach significance in the overall cohort or when the cohorts where stratified into specific NSCLC subtypes (Figure 1C). These results however, reflect what was observed previously in other studies involving lung cancer (Zhang et al., 2006; Li et al., 2013). We did not however, examine the mutational status of our patient cohort. As IL-23 can mediate STAT3 and PI3K signaling pathways amongst others, it is perhaps possible that constitutive activated pathways seen in NSCLC patients harboring these mutations, may be at increased risk of several cross talk feedback mechanisms, further perpetuating an oncogene addiction state. This may result in increased levels of IL-23R. To our knowledge at present there is no existing data in the literature which has determined whether NSCLC mutational status alters or affects the IL-23/IL-23R axis.

IL-23R levels were significantly induced (*p* < 0.05) following HDi treatment in the both an adenocarcinoma (A549) and a squamous cell carcinoma (SK-MES-1) cell line (Figure 2). Using ChIP, we confirmed that HDi acts by directly remodeling the IL-23R promoter region (Figure 3). As is demonstrated in Figure 3, HDi treatment results in increased acetylation on histones H3 and H4 at the IL-23A promoter, along with other post-translational modifications associated with either transcriptionally active chromatin or at poised promoters (Berger, 2007; Pekowska et al., 2010). HDACs and Lysine demethylases (KDMs) are both linked with the regulation of gene transcription, and HDi can inhibit lysine demethylases causing increased H3K4me2 (Lee et al., 2006; Huang et al., 2011), in agreement with the results observed by us at the IL-23R promoter (Figure 3).

Treatment of the A549 cell line with a DNMTi (DAC) resulted in a significant up-regulation of IL-23R (*p* < 0.05, Figure 4), indicating that in addition to histone post-translational modifications, DNA CpG methylation at the promoter region is also involved in the regulation of this gene. Treatment with the chemotherapeutic agent, Gemcitabine, also induced the expression of IL-23R (*p* < 0.05, Figure 5). We have previously shown that Gemcitabine is a potential DNMTi (Gray et al., 2012).

Finally, we treated NSCLC cells with a small molecule capable of inhibiting IL-12/IL-23 production. Cells were treated with Apilimod for a period of 72 h, after which time the proliferative capacity of the cells was determined (Figure 6). The adenocarcinoma cell line (A549) displayed enhanced sensitivity to the compound compared to the SK-MES-1 cell line, although apilimod caused significant decreases in both cell lines overall (Figure 6). An approximate 50% reduction in A549 proliferation was evident at 0.1 μM, however a 50% decrease was not reached in the SK-MES-1 cell line even at the highest dose of 1 mM. These differential responses may in part be due to differential receptor expression, as both cell lines robustly express IL-12Rβ1, however IL-23R is only expressed in the A549 cell line (Baird et al., 2013). We and others have shown that IL-23R levels can affect a cell line’s response to IL-23 (Baird et al., 2013; Li et al., 2013; Shen et al., 2013) and this would appear to translate to differential responses to Apilimod. Various splice isoforms of IL-23R have also been identified and differ between NSCLC subtypes, which may also affect the response to Apilimod (Zhang et al., 2006). It may however be possible to use IL-23R expression as a candidate biomarker to predict responsiveness to Apilimod therapy.

The IC50 values for the lung cell lines are of a much higher magnitude than that seen for PBMC, where an IC50 of 10 nM was observed (Wada et al., 2007), and may possibly be due to the aggressive nature of lung cancer cells. Nevertheless, a recent Phase 2a multi-center, open-label clinical trial has completed in psoriasis patients with the predominant focus on biomarker-based measures of biological response (Wada et al., 2012). In this study patients received doses of Apilimod up to 70 mg once daily. The maximum Apilimod concentration observed in plasma at this dose was 265 ± 183 nM 2 h post-dose (Wada et al., 2012). It would appear that 70 mg (twice daily) is the maximum dose tolerable as CNS-related adverse events (headache, flushing, hypoesthesia, dizziness, and paresthesia) were observed at a 105 mg BID dose level in a Phase I study (Wada et al., 2012). As we observe a significant decrease in cellular proliferation in our NSCLC cell lines at concentrations as low as 100 nM, particularly for those cells that express significant levels of IL-23R (A549), potential clinical efficacy may be achieved using this dose.

Based on our results, IL-23R is expressed in a set of primary NSCLC tumors. The expression of IL-23R in NSCLC was found to be dynamically regulated both by chemotherapy drugs and epigenetic mechanisms. These results may, therefore, have important implications for treating patients with epigenetic targeting therapies and/or Gemcitabine. Our results indicate that HDAC inhibitors may not be a viable therapeutic treatment option in NSCLC, at least as a single entity given that in our experience, both SAHA and TSA increase the expression of both IL-23 and IL-23R levels. Combinational studies would be warranted to determine if HDACi therapy would be more valuable in combination with Apilimod.

The results presented here may have implications for the differential treatment of adenocarcinoma and squamous cell carcinoma NSCLC patients. Although Gemcitabine is usually given in a combination therapy with cisplatin for squamous cell carcinoma patients, our study has shown that Gemcitabine increases the levels of IL-23R in both NSCLC subtypes. Therefore should the IL-23 axis prove to be a prognostic marker in NSCLC, Gemcitabine may not be therapeutically advantageous for squamous cell carcinoma patients. Apilimod may however be a beneficial treatment option for NSCLC patients that express a functional IL-23R, given that it results in significant decreases in proliferation in the A549 adenocarcinoma cell line which expresses higher levels of the IL-23R than the SK-MES-1 cell line. It is possible that IL-23R levels may represent a novel potential biomarker for sensitivity to Apilimod. We have not examined the expression levels in additional adenocarcinoma and squamous cell carcinoma NSCLC cell lines. However, in the paper by Lin and colleagues (Li et al., 2013) IL-23R receptor expression levels in A549 and SK-MES, were similar to our observations, confirming our data. They also examined levels IL-23R receptor levels in a large cohort of patient samples (*n* = 137). According to their data, IL-23R positivity was observed in 85–90% of adenocarcinomas and small cell carcinomas, while IL-23R positivity was only observed in 7.5% of squamous cell carcinomas (Li et al., 2013 supplementary Table 2).

There also exists the possibility that combinatorial treatments with Apilimod and standard chemotherapies may have additive or synergistic effects. To our knowledge, there is currently no data available examining the effect of cisplatin or other platinum agents on IL-23R expression in lung cancer. However as platinum based agents have been shown to increase the expression of a number of inflammatory mediators such as NF-kB, it is therefore conceivable that cisplatin based therapy may increase the expression of IL-23/IL-23R. Currently we are examining IL-23 and IL-23R expression levels in a panel of isogenic of parent (sensitive) and resistant NSCLC cell lines generated in our unit (Barr et al., 2013) with a view to conducting combination treatments of Apilimod and cisplatin in future studies.

Further study is needed to elucidate the clinical impact of targeting the IL-23/IL-23R axis in NSCLC. A larger IHC study will be required to fully correlate expression levels with clinico-pathological data and to determine if IL-23R levels can act as a biomarker for sensitivity to anti-IL-23 mediated therapies.

Finally, to our knowledge, this is the first study to examine the potential utility of Apilimod in a cancer setting where we have shown that this compound can significantly reduce the proliferative rate of NSCLC cells. It has recently been shown that levels of IL-23 are elevated in breast cancer (Qian et al., 2013b). Although currently in clinical trials for auto-immune conditions, it may be therefore be worthwhile to further examine the efficacy of this compound in the treatment of lung cancer and other cancers.

## Conflict of Interest Statement

The authors declare that the research was conducted in the absence of any commercial or financial relationships that could be construed as a potential conflict of interest.
